# Management of Postoperative Pancreatic Fluid Collection and Role of Endoscopy: A Case Series and Review of the Literature

**DOI:** 10.3390/diagnostics15101258

**Published:** 2025-05-15

**Authors:** Chiara Coluccio, Ilaria Tarantino, Maria Chiara Petrone, Edoardo Forti, Stefano Francesco Crinò, Alessandro Fugazza, Roberto Di Mitri, Cecilia Binda, Davide Trama, Arnaldo Amato, Alessandro Redaelli, Germana De Nucci, Fabia Attili, Mario Luciano Brancaccio, Claudio Giovanni De Angelis, Mauro Lovera, Antonio Facciorusso, Andrea Anderloni, Carlo Fabbri

**Affiliations:** 1 Gastroenterology and Digestive Endoscopy Unit, Forlì-Cesena Hospitals, AUSL Romagna, 47121 Forlì-Cesena, Italy; 2 Endoscopy Service, Department of Diagnostic and Therapeutic Services, IRCCS-ISMETT, 90100 Palermo, Italy; 3 Pancreato-Biliary Endoscopy and Endosonography Division, Pancreas Translational and Clinical Research Center, San Raffaele Scientific Institute IRCCS, Vita Salute San Raffaele University, 20132 Milan, Italy; 4 Digestive and Interventional Endoscopy Unit, Ospedale Ca’ Granda Niguarda, 20162 Milan, Italy; 5 Department of Medicine, Diagnostic and Interventional Endoscopy of the Pancreas, The Pancreas Institute, University Hospital of Verona, 37134 Verona, Italy; 6 Division of Gastroenterology and Digestive Endoscopy, Department of Gastroenterology, IRCCS—Humanitas Research Hospital, 20089 Milan, Italy; 7 Gastroenterology and Endoscopy Unit, “ARNAS Civico-Di Cristina-Benfratelli” Hospital, 90127 Palermo, Italy; 8 Department of Gastroenterology, Valduce Hospital, 22100 Como, Italy; 9 Endoscopy Unit, San Gerardo Hospital, 20900 Monza, Italy; 10 Gastroenterology and Endoscopy Unit, ASST Rhodense, Garbagnate Milanese, 20024 Milan, Italy; 11 Endoscopia Digestiva, Dipartimento di Scienze Mediche e Chirurgiche, Fondazione Policlinico Universitario A. Gemelli IRCCS, 00168 Rome, Italy; 12 Division of Gastroenterology, S. Maria delle Croci Hospital, 48121 Ravenna, Italy; 13 Gastroenterology and Digestive Endoscopy Unit, AOU Città della Salute e della Scienza, University of Turin, 10126 Turin, Italy; 14 Digestive Endoscopy Unit, Fondazione Poliambulanza Istituto Ospedaliero, 25133 Brescia, Italy; 15 Gastroenterology Unit, Department of Experimental Medicine, Università del Salento, 73100 Lecce, Italy; 16 Gastroenterology and Endoscopy Unit, Fondazione IRCCS Policlinico San Matteo, 27100 Pavia, Italy

**Keywords:** postoperative fluid collection (POFC), postoperative pancreatic fluid collection (PPFC), endoscopic ultrasound-guided drainage (EUS-D), pancreatic fluid collection (PFC), percutaneous drainage (PCD)

## Abstract

**Background:** Postoperative fluid collections (POFCs) after abdominal surgeries, particularly pancreatic surgeries, are associated with high morbidity and mortality rates and were historically managed with surgical re-exploration and drainage. In particular, postoperative pancreatic fluid collections (PPFCs) are the most common complications after pancreatic surgery resulting from pancreatic leaks. They occur in up to 50% of cases, and approximately 10% of them need to be drained to avoid further sequelae. Endoscopic ultrasonography (EUS)-guided drainage of PPFCs represents the first-line treatment nowadays, but many aspects are still debated. **Methods:** We describe a retrospective case series of patients from multiple Italian centers who underwent EUS-guided drainage (EUS-D) of POFCs, aiming to provide data on the efficacy and safety of this procedure, supported by a review of the existing literature on this topic. The primary outcomes were technical and clinical success, and the secondary outcomes were the type and rate of adverse events (AEs) and the rate of recurrence. **Results:** A total of 47 patients were included. The procedure demonstrated a technical success rate of 98% (46/47) and a clinical success rate of 96% (45/47). The rate of AEs was 11% (5/47), represented by bleeding (3/5), stent occlusion (1/5), and buried syndrome (1/5). **Conclusions:** Management of POFCs has shifted over time towards an endoscopic approach with optimal efficacy and safety.

## 1. Introduction

Postoperative fluid collections (POFCs) are one of the major causes of morbidity and mortality after abdominal surgery [1]. Even if they are usually asymptomatic, many cases have been reported to cause severe pain, gastric outlet obstruction, fistulas, intraabdominal infection, and sepsis, with mortality rates of undrained POFCs ranging from 45% to 100% [2,3]. The incidence of POFCs varies depending on the type of surgery, with pancreatic surgery being one of the most common procedures associated with POFCs. In particular, postoperative pancreatic fluid collections (PPFCs) are significant and relatively common adverse events following pancreatic surgery, with incidence rates varying depending on the specific procedure [1,4]. For instance, up to 25% of patients undergoing pancreaticoduodenectomy may develop these collections, and this rate can increase to 43% following distal pancreatectomy and ranges from 20 to 60% after central pancreatectomy and enucleation [4].

PPFCs are caused by the leakage of pancreatic juice from anastomoses [5,6], and they are classified by the International Study Group on Postoperative Pancreatic Fistula (ISGPF) based on their severity and the required interventions. The ISGPF categorizes postoperative pancreatic fistulas (POPFs) and PPFCs that resolve spontaneously as Grade A or biliary leakage, PPFCs that require persistent drainage as Grade B, and PPFCs that require reoperation and cause organ failure or death as Grade C [7,8].

Mutignani et al. have also identified three distinct types of pancreatic injury leading to pancreatic leaks/fistulas, according to the anatomic position of the leak and the injured duct (the main pancreatic duct or small branch duct). This is an endoscopy-oriented classification, and leak types are classified into three categories: type I (leakage from the small side branches or the very distal end of the pancreatic duct); type II (leakage from the main pancreatic duct, with disconnected/disrupted pancreatic duct syndrome); and type III (leakage after pancreatectomy), which is divided into type IIIP (after distal pancreasectomy) and type IIID (after duodenocephalopancreasectomy) [9].

Most POFCs are asymptomatic, and they are usually managed conservatively with fasting, total parenteral nutrition, antibiotics, and/or somatostatin analogs. If symptoms related to the collection or complications occur, there is an indication for drainage, and this happens in just 10% of cases [10,11].

Historically, PPFCs were managed with surgical re-exploration and drainage [12,13].

In recent years, endoscopic treatment options, particularly the use of endoscopic ultrasound (EUS) and endoscopic retrograde cholangiopancreatography (ERCP), have gained more and more popularity for the management of PPFCs [14,15,16].

Here, we present a large, retrospective, multicenter case series of patients with POFCs managed by endoscopic ultrasound-guided drainage (EUS-D) with the aim of reporting its efficacy and safety, accompanied by a comprehensive literature review on the subject.

## 2. Materials and Methods

In 2019, the Interventional Endoscopy and Ultrasound (i-EUS) group was created in Italy with the goal of supporting and promoting educational initiatives to optimize the clinical use of lumen-apposing metal stents (LAMSs). The group comprises centers from across Italy, each with varying levels of expertise in interventional endoscopy. A level of expertise was determined based on the annual volume of EUS and ERCP procedures, the total number of LAMS placements, and the type of indication for LAMS placement. Although no formal standardization of procedural protocols was enforced due to the retrospective nature of the study, all participating centers adhered to widely accepted national guidelines and were part of the i-EUS group, ensuring a minimum level of procedural uniformity. Centers with less expertise were defined as those that performed fewer than 250 EUSs/year and fewer than 200 ERCPs/year at the time of the study and had placed an overall number of LAMSs < 20. All centers in the i-EUS group that showed interest in participating were included in a multicenter, retrospective data collection involving all procedures of EUS-D with LAMSs for three major indications (PFCs and gallbladder and biliary indications) [17].

This retrospective, multicenter case-series analysis included 13 Italian secondary and tertiary endoscopy units that performed EUS-guided procedures, including EUS-D for POFCs.

The study aimed to evaluate the efficacy and safety of EUS-D as a treatment for POFCs. The period of study spanned eight years, from January 2013 to December 2020, and data were collected regarding demographics, imaging performed prior to stent placement, the type and size of POFCs, the indications for collection drainage, the stent type, and any adverse events during or after the procedure.

The inclusion criteria were as follows: patients aged > 18 years; patients’ ability to sign an informed consent form for the procedure; patients with a confirmed diagnosis of POFC based on imaging studies (CT or MRI); presence of symptoms related to fluid collection, such as abdominal pain, early satiety, or signs of infection (e.g., fever or elevated white blood cell count); collections that were anatomically accessible via EUS and deemed appropriate for drainage by the treating physician. The exclusion criteria were as follows: patients aged < 18 years or not able to provide an adequate informed consent; fluid collections deemed asymptomatic, which could be managed conservatively.

### 2.1. Patient Selection

The present retrospective case-series study included patients diagnosed with POFCs following abdominal surgery. Patients were selected based on biochemical and imaging evidence of POFCs along with the presence of clinical indications for drainage, such as symptoms of infection, abdominal pain, or signs of gastric outlet or biliary obstruction.Imaging techniques were useful in both the diagnosis and subsequent management of POFCs. Prior to the procedure, most patients underwent a contrast-enhanced CT scan to assess the size, location, and complexity of the fluid collection. In cases where CT was contraindicated or insufficient detail was provided, an MRI was performed. MRI was particularly useful in evaluating the presence of internal necrosis and in characterizing the fluid content of the collections, which could influence the choice of drainage technique and stent. Patients were monitored using a combination of clinical assessments (in terms of pain, fever, and signs of infection), laboratory tests (including white blood cell count, serum amylase/lipase levels, and C-reactive protein/procalcitonin), and imaging during follow-up.

### 2.2. Technique

The EUS-guided drainage procedures were performed using linear-array echoendoscopes and carbon dioxide insufflation; patients were under deep sedation or general anesthesia at the discretion of the anesthesiologist. The choice of stent was at the discretion of the endoscopist, based on collection characteristics and the operator’s experience and availability. Two main techniques were used for accessing the collections:

- Single-Stage Procedure: An electrocautery-enhanced lumen-apposing metal stent (LAMS) was inserted directly into the PPFC under EUS guidance. The use of electrocautery allowed for simultaneous tissue penetration and stent deployment, simplifying the procedure and reducing the risk of complications. This technique was particularly favored for its ability to streamline the process and minimize the number of steps required for successful stent placement.
- Needle-Plus-Guidewire Technique: A stepwise approach in which, initially, a 19-gauge needle was used to puncture the collection under EUS guidance. After successful puncture, a 0.035-inch guidewire was introduced through the needle and looped within the collection to provide stability. The puncture site was then enlarged using a cystotome and, over a guidewire, a covered metal stent or double-pigtail plastic stent (DPPS) was deployed.

### 2.3. Endpoints

**Figure 1:**
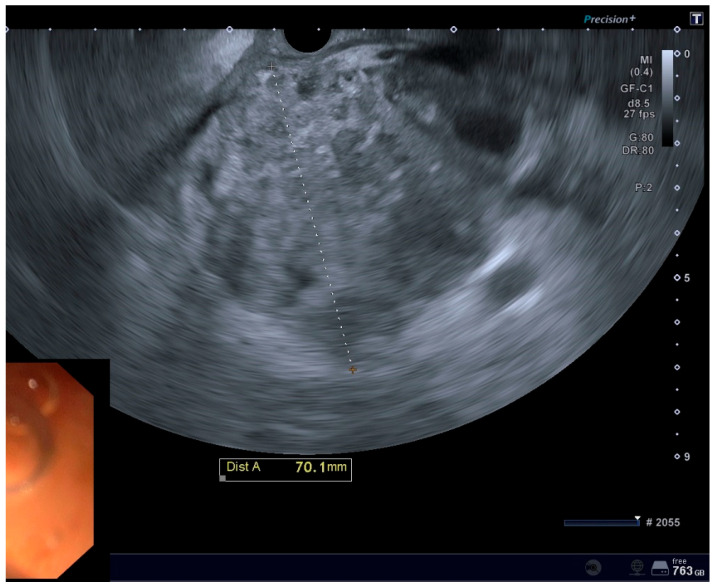
EUS imaging of a POFC.

The primary endpoint of this study was to assess the rate of technical and clinical success of EUS-D of POFCs (Figure 1). Technical success was defined as the successful placement of a stent inside the collection; clinical success was defined as a reduction in the size of the walled-off necrosis (WON) or pseudocyst to less than 2 cm on axial imaging (range: 2 weeks to 6 months) following stent placement, without the need for additional endoscopic or surgical interventions.

Secondary endpoints were as follows: type and rate of adverse events (AEs), with severity graded by the ASGE lexicon [18], and rate of POFC recurrence, defined as the reappearance of a collection or symptoms after initial successful treatment. Recurrence rates were assessed to determine the long-term efficacy of EUS-guided drainage.

### 2.4. Statistical Analysis

The data were entered into an Excel database as soon as they were submitted by the participating centers, ensuring patient anonymity. This process allowed for real-time data collection while maintaining anonymity to comply with privacy and data protection regulations. Descriptive statistics for nonparametric distribution were used. Means, medians, and standard deviations were used to report the results, as appropriate.

## 3. Results

The series included a total of 47 patients (38% women and 62% men) from 13 Italian centers, and most POFCs were caused by pancreatic surgeries. The patients and their clinical characteristics are outlined in Table 1.

In 40 patients (87%), a CT scan was performed prior to the drainage; in 4 patients (9%), an MRI scan was performed; and in 3 cases (3%), both scans were performed.

Among all the included patients, 19 (40%) were identified as having walled-off necrosis (WON), while the remaining patients were characterized by pseudocysts, which are typically more homogeneous and without a significant amount of necrotic debris. POFCs were mainly located near the pancreatic body (*n* 33, 70%), the rest at the level of the tail (*n* 11, 23%) or the head (*n* 3, 6%). Most of the collections were uniloculated (*n* 38, 81%).

The mean size of the POFCs on EUS evaluation was 88.8 mm in width (SD 61.8) and 77.2 mm in length (SD 45.7).

Procedural and technical characteristics are outlined in Table 2.

**Figure 2:**
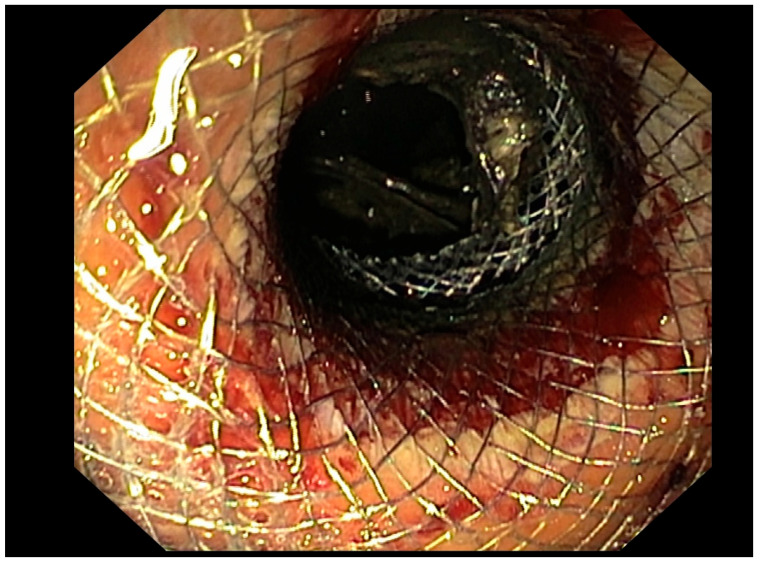
Use of a LAMS for EUS-D of POFC.

The most common type of access to the collection was the single-stage procedure (29/47, 62%), and the Hot Axios stent was used in all cases (*n* 29/29, 100%) (Figure 2).

Eighteen POFCs (18%) were drained using the needle-plus-guidewire technique, and the stents utilized were as follows: the Axios stent (*n* 1/18, 6%), the Spaxus stent (*n* 1/18, 6%), the double-pigtail plastic stent (DPPS) (*n* 1/18, 6%), the Nagi stent (*n* 10/18, 55%), and the Hanarostent (*n* 5/18, 28%).

The majority of the procedures (*n* 44,94%) were performed using a transgastric approach, while in a smaller number of cases (*n* 3, 6%) a transduodenal approach was used, this approach typically being reserved for collections located near the head of the pancreas or when the transgastric approach is not feasible.

Among six patients who underwent positioning of electrocautery LAMSs, four patients also received one 10 Fr pigtail stent, one patient received an 8 Fr pigtail stent, and one patient received a 7 Fr pigtail stent.

Technical success was achieved in 46/47 patients (98%). The only patient in whom technical success was not achieved, because of technical complexity, was subsequently surgically treated. The mean hospital stay following stent placement was 17 days (SD 22).

Clinical success was achieved in 45/47 patients (96%) (Table 3). No patients needed concomitant percutaneous drainage.

In some cases, additional interventions were required, such as direct endoscopic necrosectomy (DEN), which was performed in a total of seven patients who did not achieve full resolution of the collection with stent placement alone, with a mean of three DEN sessions (SD = 2.6 sessions); in detail, one patient needed six sessions, three patients needed two sessions each, and the remaining three patients needed one session each.

In 34 patients (72%), the stent was removed, with a mean time between the stent positioning and removal of 48.9 days (SD = 64).

The rate of AEs was 11% (5/47). The most common AE was bleeding, which occurred in 60% of cases (3/5), followed by stent occlusion (1/5, 20%) and buried stent syndrome (1/5, 20%). One case of bleeding, graded as severe, occurred 19 days after the procedure and was managed by angiographic embolization. One of the other two cases of bleeding was moderate and resolved with stent replacement, and the other was self-limiting. The case of buried stent syndrome was managed conservatively, while the case of stent occlusion, which occurred 20 days after the stent placement, was treated by placing two pigtail stents within the LAMS.

Among the five patients who experienced adverse events, two had collections with necrotic contents, which are known to be associated with higher procedural complexity and risk. Additionally, one of the procedures was performed at a center with lower procedural volumes, which may reflect limited operator experience.

## 4. Discussion

The present study confirms that EUS-D for POFCs has a high clinical and technical success rate, in line with the current literature on this specific topic, with acceptable complication rates.

POFCs remain a major cause of postoperative morbidity, particularly following pancreatic surgery. They can cause symptoms such as severe pain and gastric outlet obstruction and can lead to complications such as infection, which is the most common and it is associated with high mortality and morbidity rates, or vascular erosion, caused by enzymatic action of pancreatic juice which erodes the walls of major arteries, causing potentially life-threatening hemorrhages. This complication, known as pseudoaneurysm, requires immediate intervention in about 9% of cases [4,17], often in the form of angiographic embolization, or, in extreme cases, emergency surgery.

Several studies have demonstrated that preoperative pancreatic duct stenting can reduce the incidence of POPFs following distal pancreatectomy, suggesting that managing pancreatic duct pressure effectively could play a crucial role in mitigating the risk of POPF formation; however, despite its theoretical benefits, prophylactic preoperative pancreatic duct stenting is not currently recommended in clinical practice due to the lack of robust evidence from well-designed, large-scale clinical trials and the potential risks associated with the procedure [19].

Historically, both surgery and PCD have been the mainstays of treatment for symptomatic POFCs. However, these methods are associated with significant drawbacks.

Repeat surgery is an invasive method associated with a high risk of morbidity and mortality, particularly in patients already weakened by a previous major surgical procedure, so it is reserved for only a select few cases. A valid treatment method is PCD, in which a catheter is inserted into the collection under ultrasound or CT guidance. Although it has shown better outcomes compared to surgical therapies [14], PCD has a success rate at first attempt of only 65%. Clear disadvantages of this option are that the tube needs to be maintained in place until the resolution of the POFC, that there are risks of an impaired quality of life and continuous external pancreatic fistulas [1,4], and that it is less successful in treating infected collections, especially in the presence of necrosis, which can lead to tube obstruction [1].

Since the first description of EUS-D of a pseudocyst by Giovannini et al., interventional EUS has expanded its indications, becoming the first-line therapy for drainage of symptomatic collections, mainly those following acute pancreatitis, the literature on which is redundant, but also for other etiologies, such as biloma, subphrenic, and pelvic abscesses [13,20], as well as POFCs, owing to its safety and efficacy [21], and our study confirms that EUS-D for POFCs has a high clinical (96%) and technical success rate (98%), in line with the current literature.

A comparative study published by Téllaz-Avila et al. on EUS-D and PCD demonstrates that EUS-D is at least as effective and safe as PCD in patients with POFCs, with comparable technical success (100% vs. 91%; *p* = 0.25), clinical success (100% vs. 84%; *p* = 0.13), recurrence (31% vs. 25%; *p* = 0.69), hospital stay-day (median 22 vs. 27; *p* = 0.35), complication (0% vs. 6%; *p* = 0.3), and mortality rates (8% vs. 6%; *p* = 0.9), with the advantage of not requiring external drainage [21].

Another recent meta-analysis by Khizar et al. showed that EUS-D is safe and efficient for PFC, whatever its etiology, with a higher clinical success rate (OR: 2.23; 95% CI: 1.45, 3.41) and a lower mortality rate (OR: 0.24; 95% CI: 0.09, 0.67) and re-intervention rate (OR: 0.25; 95% CI: 0.16, 0.40) compared with PCD [22].

Traditionally, the EUS-D of POFCs has relied on tools originally developed for EUS-guided fine-needle aspiration (FNA) and the use of wires and DPPSs designed for ERCP [23]. Although DPPSs offer some advantages, such as reducing the risk of stent migration and being relatively cost-effective, their use presents several significant limitations [24,25].

One of the primary challenges associated with DPPSs is their small stent diameter, which makes them particularly susceptible to occlusion. Moreover, the placement of these stents is technically demanding, as it requires repeated wire access across the collection, a process that is not only time-consuming but also increases the procedural complexity for the physician.

In addition, draining POFCs using DPPSs may involve the placement of multiple stents to achieve adequate drainage, especially in cases of WON [26], and achieving resolution in such cases often requires multiple revisions and stent exchanges, further adding to the procedural burden and increasing the overall risk of complications.

Theoretically, the wider lumen of the LAMS has been thought to offer a faster resolution compared with plastic stents, with a better infection control; however, no difference in clinical efficacy between the two methods has been established [27].

To the best of our knowledge, only two randomized studies have been conducted to compare the outcomes of LAMSs and DPPSs.

The first one was published in 2019 by Bang et al., and it failed to demonstrate any significant difference in terms of clinical or technical success, including the number of procedures needed [28]. The second and more recent study was published in 2023 by Kastensen et al., which included only large WON (>15 cm) affecting 42 patients. The results showed that LAMSs were not superior to DPPSs in terms of clinical success, the need for necrosectomy, length of hospital stay, or AE rates. Both techniques proved effective, with an overall mortality rate of 5%. The study concluded that DPPSs remain a viable alternative, particularly in resource-limited settings [27].

Most of the patients in our study underwent electrocautery-enhanced LAMS placement (*n* 29/47, 62%), likely due to its ease of use and efficiency, demonstrating a good clinical efficacy and safety, with a technical success rate of 100% (*n* 30/30), a clinical success rate of 97% (*n* 29/30), and an AE rate of 13% (*n* 4/30), most of these events being graded as mild (*n* 3/4, 75%), in line with the current literature.

Moreover, data from the literature support the use of LAMSs for WON, due to the possibility of allowing entry into the cyst cavity and performing DEN and intensive lavage as well [29,30]. Seven patients in our study underwent at least one necrosectomy session due to the presence of solid debris in the collection.

The rate of overall AEs in our study was 11%, with no evidence of fatal ones. Most of the AEs occurred during the endoscopic procedure, and their severity was mostly mild (only one was severe). Bleeding was the most common complication, in line with the rate of AEs for this specific condition in the most recent studies. One patient experienced buried stent syndrome, a relatively rare complication in which the stent becomes embedded in tissue and is difficult to remove. This condition can potentially lead to chronic infection or abscess formation and may require surgical intervention to resolve it, as reported in our study. While the sample size did not allow for a robust multivariate analysis, these observations highlight the importance of both anatomical and procedural factors in influencing complication rates. Patient characteristics, such as the presence of necrotic tissue, and center-related variables, such as procedural volume, should be carefully considered when planning EUS-guided drainage. Future prospective studies should aim to identify predictors of complications through larger cohorts and stratified data analysis.

EUS-D, while offering numerous benefits, has its own limitations and challenges; first of all, there is the technical complexity of the procedure, which requires a high level of expertise on the part of the endoscopist, influencing the rate of positive procedural outcomes, particularly in complex cases involving large or multiloculated collections, or collections located in difficult-to-access areas, such as far away from the gastrointestinal wall [31].

The success of EUS-guided drainage is closely linked to the operator’s experience, as it involves technically demanding procedures that require specific training and expertise. In our study, participating centers belonged to the i-EUS group and included secondary and tertiary endoscopy units, reflecting variability in operator skills. This variability may have influenced procedural decisions and outcomes. However, it also provides a realistic representation of clinical practice across different healthcare settings.

Another issue is the higher procedural costs compared to PCD. Despite these aspects, EUS-D offers several important advantages, such as the ability to visualize anatomically relevant structures, such as surrounding organs and vessels involved in a collection. In addition to its technical feasibility and safety, EUS-D has also been shown to improve quality of life and reduce the risk of infection. Even in patients with infectious POFCs, draining the fluid into the stomach via EUS-D did not result in fever, gastroenteritis, or retrograde infection. Furthermore, EUS-D prevents fluid and electrolyte loss, which can occur after PCD, and reduces the risk of persistent collections and fistulas [14].

Patients who do not respond to minimally invasive endoscopic or radiologic treatments are usually managed surgically. However, emerging endoscopic approaches using agents like N-Butyl-2-cyanoacrylate (NBCA)—a non-biologic glue already used in vascular and gastrointestinal procedures—combined or not with a metallic coil to prevent glue migration have shown promise in closing pancreatic fistulas [32,33]. Although based on limited data, this combined method could offer a viable alternative for challenging cases.

While our study provides valuable insights into the management of POFCs, certain limitations must be acknowledged. The retrospective nature of the study may have introduced selection bias. Additionally, the absence of a control group, such as patients treated with alternative approaches like percutaneous drainage (PCD) or surgical re-intervention, prevented a direct comparison of outcomes. As a result, while the study provides valuable real-world data on the safety and efficacy of EUS-guided drainage, it does not allow conclusions about its relative effectiveness compared to other standard treatments. To establish such comparisons reliably, prospective, controlled trials are needed.

Furthermore, our study did not provide data regarding the timing of EUS-D, which still remains an open issue [4], in contrast to PFCs after acute pancreatitis, for which a lot of evidence has been created. Storm et al. compared clinical outcomes in acute (<2 weeks), early (<4 weeks), and delayed EUS-D for POFCs and found no significant differences in clinical success (95%, 93%, and 94%, respectively) or AEs (21.4%, 15.0%, and 30.3%, respectively) [34]. Oh et al. also evaluated clinical outcomes according to the presence of encapsulation, in addition to the timing of EUS-D. Technical success, clinical success, and AE rates were also similar between early and delayed interventions [35]. It is fair to say that, while encapsulation is emphasized in PFCs after acute pancreatitis to determine the timing of interventions, it is still unclear whether encapsulation of POFCs might favorably affect the clinical outcomes, especially the safety, of early drainage, and further prospective studies are needed to evaluate the appropriate timing.

Data are also lacking regarding the role of ERCP in managing POFCs. ERCP can also be an option for POFCs due to pancreatic disruption or leakage after distal pancreatectomies, but it is less frequently reported than EUS-D [4]. Treatment options are represented by pancreatic sphincterotomy, nasopancreatic duct tube placement, plastic stent placement, or a combination of these. Furthermore, conventional ERCP is not always technically possible owing to the surgically altered anatomy (e.g., after duodenocephalopancreasectomy), and balloon enteroscope-assisted ERCP (BE-ERCP) is necessary, but data on BE-ERCP are limited and the technical success rates of BE-ERCP for pancreatic indications are not as high as those for biliary indications. Thus, expertise in both BE-ERCP and EUS-guided pancreatic duct drainage is important for managing cases after pancreaticoduodenectomy [4].

While our cohort represents one of the largest multicenter Italian studies specifically focused on EUS-guided drainage of POFCs, we acknowledge that the relatively small sample size may limit the generalizability of our findings. Future prospective studies with larger patient populations are needed to validate and expand upon our conclusions. Further and more comprehensive studies are needed to strongly confirm EUS-D as a first-line therapy for POPFs and to balance the pros and cons.

## 5. Conclusions

This study provides an extensive overview of the efficacy and safety of EUS-D of POPFs, contributing to the growing body of literature supporting its use as a first-line treatment. Given its promising results, further research is warranted to optimize the technique and expand its applications. Future studies could focus on comparing different types of stents in terms of their efficacy and safety, exploring the optimal timing for stent placement and removal, and investigating the long-term outcomes.

## Figures and Tables

**Table 1:** Baseline characteristics of included patients.

Age, mean (SD), years	61.7 (15.1)
Sex, female, %	38%
Indication for POFC drainage, *n*, (%)	
▪ Symptoms suggestive of infected collection	22 (47%)
▪ Abdominal pain	13 (28%)
▪ Early satiety	5 (11%)
▪ Vomiting	4 (8%)
▪ Gastric outlet obstruction	2 (4%)
▪ Vessel thrombosis	1 (2%)
Location of POFC, *n*, (%)	
▪ Head	3 (6%)
▪ Body	33 (70%)
▪ Tail	11 (23%)
Mean size of POFCs on EUS evaluation, width, mm	88.8 mm
Mean size of POFCs on EUS evaluation, length, mm	77.2 mm
Type of POFC, *n*, (%)	
▪ WON	19 (40.4%)
▪ Pseudocyst	28 (59.6%)
Multiloculated POFC, *n*, (%)	9 (19%)
Uniloculated POFC, *n*, (%)	38 (81%)

**Table 2:** Procedural characteristics.

Type of access to the collection, *n*, (%)	
▪ Single-stage procedure	29/47 (62%)
Hot Axios stent	29/29 (100%)
▪ Needle-plus-guidewire technique	18/47 (38%)
Hanarostent	5/18 (28%)
Nagi stent	10/18 (54%)
Plastic stent	1/18 (6%)
Spaxus stent	1/18 (6%)
Hot Axios stent	1/18 (6%)
Type of approach	
▪ Transgastric approach	44/47 (94%)
▪ Transduodenal approach	3/47 (6%)
Patients who required placement of DPPSs within LAMSs, *n*	6/46 (13%)
▪ 10 Fr double-pigtail	4/46 (9%)
▪ 8 Fr double-pigtail	1/46 (2%)
▪ 7 Fr double-pigtail	1/46 (2%)

**Table 3:** Outcomes and AEs.

Clinical success, *n* (%)	45/47 (96%)
Technical success, *n* (%)	46/47 (98%)
PPFC recurrence	1/47 (2%)
▪ Type of treatment after recurrence	Surgery
Number of patients who needed necrosectomy to achieve full resolution of the collection	7/46 (15%)
Rate of adverse events, *n* (%)	5/47 (11%)
Type of adverse event, *n* (%)	
▪ Bleeding	3/5 (60%)
▪ Stent occlusion	1/5 (20%)
▪ Buried stent syndrome	1/5 (20%)

## Data Availability

The data presented in this study are available on request from the corresponding author.
